# *In Vitro* and *Ex Vivo* Analysis of *CHRNA3* and *CHRNA5* Haplotype Expression

**DOI:** 10.1371/journal.pone.0023373

**Published:** 2011-08-12

**Authors:** Glenn A. Doyle, Min-Jung Wang, Andrew D. Chou, John U. Oleynick, Steven E. Arnold, Russell J. Buono, Thomas N. Ferraro, Wade H. Berrettini

**Affiliations:** 1 Center for Neurobiology and Behavior, Department of Psychiatry, University of Pennsylvania, Philadelphia, Pennsylvania, United States of America; 2 Johnson and Johnson, Malvern, Pennsylvania, United States of America; 3 Research Service, Department of Veterans Affairs Medical Center, Coatesville, Pennsylvania, United States of America; Niels Bohr Institute, Denmark

## Abstract

Genome-wide association studies implicate variations in *CHRNA5* and *CHRNA3* as being associated with nicotine addiction (NA). Multiple common haplotypes (“risk”, “mixed” and “protective”) exist in Europeans; however, high linkage disequilibrium between variations in *CHRNA5* and *CHRNA3* makes assigning causative allele(s) for NA difficult through genotyping experiments alone. We investigated whether *CHRNA5* or *CHRNA3* promoter haplotypes, associated previously with NA, might influence allelic expression levels. For *in vitro* analyses, promoter haplotypes were sub-cloned into a luciferase reporter vector. When assessed in BE(2)-C cells, luciferase expression was equivalent among *CHRNA3* haplotypes, but the combination of deletion at rs3841324 and variation at rs503464 decreased *CHRNA5* promoter-derived luciferase activity, possibly due to loss of an SP-1 and other site(s). Variation within the *CHRNA5* 5’UTR at rs55853698 and rs55781567 also altered luciferase expression in BE(2)-C cells. Allelic expression imbalance (AEI) from the “risk” or “protective” haplotypes was assessed in post-mortem brain tissue from individuals heterozygous at coding polymorphisms in *CHRNA3* (rs1051730) or *CHRNA5* (rs16969968). In most cases, equivalent allelic expression was observed; however, one individual showed *CHRNA5* AEI that favored the “protective” allele and that was concordant with heterozygosity at polymorphisms ∼13.5 kb upstream of the *CHRNA5* transcription start site. Putative enhancer activity from these distal promoter elements was assessed using heterologous promoter constructs. We observed no differences in promoter activity from the two distal promoter haplotypes examined, but found that the distal promoter region strongly repressed transcription. We conclude that *CHRNA5* promoter variants may affect relative risk for NA in some heterozygous individuals.

## Introduction

Nicotine is the main addictive agent in cigarette smoke [1]. Nicotine addiction (NA) can be thought of in terms of an “imbalance" between the rewarding and aversive effects of nicotine self-administration (through cigarette smoking). Nicotinic acetylcholine receptors (nAChR) are a family of hetero- and homo-pentameric protein complexes that form ligand-gated ion channels found at the neuromuscular junctions and in the central nervous system. When activated by acetylcholine, or other agonists such as nicotine, the nAChRs allow influx of Ca^2+^ and other ions into neurons. A variety of nAChRs are expressed in brain, including (α4β2)_2_* (where * indicates differential incorporation of subunits such as β2, α4, or α5) subtypes thought to be the major high affinity brain receptor for nicotine; however, other subtypes including those containing the α5, α3 and β4 subunits are also expressed in brain [2]. Importantly, those that contain the α3 and α5 subunits are expressed in brain regions involved in reward, emotion, learning and memory (nucleus accumbens, amygdala and prefrontal cortex) as well as in regions involved in aversion (medial habenula). Studies in mice implicate (α4β2)_2_* nAChRs in behaviors related to nicotine addiction [3], [4]. Moreover, a recent study of α5 subunit knockout mice revealed the importance of α5 containing nAChRs in nicotine self-administration through rescue of α5 expression in the medial habenula [5]. Further, the utility of varenicline (a partial agonist at (α4β2)_2_* nAChRs) in smoking cessation in humans [6] also demonstrates the importance of (α4β2)_2_* nAChRs in human NA.

Numerous genetic studies in human populations implicate a role for the *CHRNA5* and *CHRNA3* genes in NA [7]–[18]. All studies identify the same alleles as increasing risk for NA, but the effect size is small (odds ratio ∼1.3, or ∼1–2 cigarettes per day in quantitative analyses). *CHRNA5* and *CHRNA3* are located in tandem on human chromosome 15 and encode the α5 and α3 nAChR subunits, respectively. These genes are in high linkage disequilibrium (LD) with one another, forming a large haplotype block [14], which complicates identification of the causative polymorphism(s) for NA.

One coding single nucleotide polymorphism (cSNP) studied extensively is a mis-sense variant in *CHRNA5* (rs16969968; G>A) that creates a D398N amino acid change, with the minor “A” allele being the NA risk allele. Comparison of the mouse (α4β2)_2_^#^ (where ^#^ indicates human α5-D398 or α5-N398) nAChRs expressed in HEK293T cells revealed similar EC_50_ values for both nAChR isoforms, but diminished maximal response for the (α4β2)_2_α5-N398 isoform when epibatidine was used as an agonist [15]. Human (α4β2)_2_α5-N398 nAChR isoforms have diminished calcium permeability and more extensive desensitization at 3 seconds after acetylcholine application compared to (α4β2)_2_α5-D398 isoforms [19]. Importantly, whereas the rs16969968 variant has a high minor allele frequency (MAF) in the European population (MAF = 0.42), it is not as prevalent in the African American (MAF = 0.04), Asian (MAFs = 0.01–0.03) and sub-Saharan African (MAF = 0.00) populations [13], [15], [20], [21]. The fact that each of these populations can exhibit NA suggests that other variants in LD with rs16969968, or loci outside the *CHRNA5-A3-B4* cluster, influence the NA phenotype in individuals of European and non-European ancestries.

**Figure 1:**
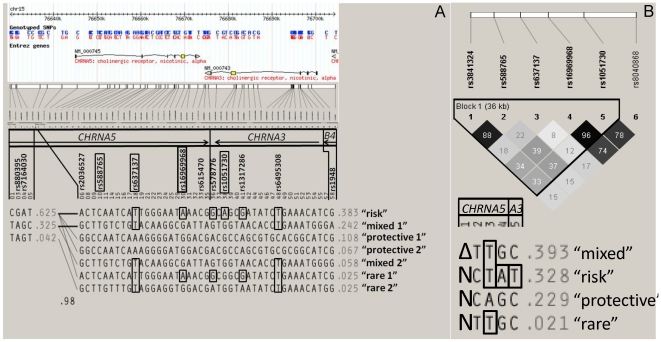
Haplotype and LD structure of *CHRNA5-CHRNA3* genes in Europeans. Haploview 4.2 [23] was used to download the version 2, release 21 CEU dataset between Chr15:76638000-76705000 from the HapMap project. The method of confidence intervals was used to generate haplotype blocks [24]. (A) The relative position of HapMap SNPs, numbered 1–58, along each gene is shown schematically at the top. Five major (>5%) and two rare (<5%) haplotypes of the gene cluster are shown with risk alleles in each haplotype associated with NA boxed in black. Those SNPs that were genotyped for haplotype analysis shown in Figure 1B are also boxed in black. The first or “risk” haplotype contains multiple SNPs associated with NA including a mis-sense coding SNP (cSNP) (rs16969968-[A]) in *CHRNA5* and a synonymous cSNP (rs1051730-[T]) in *CHRNA3* (Note: The alleles for *CHRNA3* are those of the opposite strand such that, for example, rs1051730 is an “A” rather than a “T”). The “mixed” haplotypes (second and fifth) contain many SNPs associated with NA, but not rs16969968-[A] or rs1051730-[T] (Note: The only difference between the second and fifth “mixed” haplotypes shown is at rs1948 in *CHRNB4*. Thus, the “mixed” haplotypes have a combined frequency of 24.2%+5.8% = 30% as defined in [14]. The third and fourth haplotypes are considered “protective,” containing no SNPs associated with NA. The sixth and seventh “rare” haplotypes also have a mixed allele structure with respect to SNPs associated with NA. Note that because it is not in the HapMap dataset, rs3841324 is not shown in the haplotype block structure shown in Figure 1A. (B) Haplotype analysis of *CHRNA5* and *CHRNA3* including rs3841324. Eighty European-Americans were genotyped at 6 polymorphisms (numbered 1–6) across the *CHRNA5* and *CHRNA3* (“*A3*”) genes. Haplotype and linkage disequilibrium (LD) analysis was done using Haploview 4.2 [23]. A solid spine of LD was used to define haplotype blocks. The three common and one rare haplotypes containing the normal ([N]) or deletion ([Δ]) allele at rs3841324 are shown with the r^2^ values of the LD plot indicated in grayscale squares. Darker squares represent greater LD between SNPs. As in Figure 1A, risk alleles are outlined with black boxes. Note the “mixed” haplotype containing rs3841324-[Δ] (39.3%) in Figure 1B is equivalent to the second or fifth “mixed” haplotype (summation of 30%, see above) shown in Figure 1A [14]. Also note that the cSNP rs8040868 in *CHRNA3* is not included with the haplotype blocks because it falls outside of the defined block 1 structure.

We report functional analysis of variants within haplotypes of *CHRNA5* and *CHRNA3* in people of European ancestry. *CHRNA5* and *CHRNA3* promoter-5′UTR haplotypes were examined *in vitro*, in the human neuroblastoma cell line (BE(2)-C) which expresses both *CHRNA5* and *CHRNA3*, to study the influence of promoter and 5′UTR polymorphisms on transcription and translation, respectively. The reward pathway is likely involved in whether an individual who initiates smoking is compelled to continue smoking. Thus, the potential for *CHRNA5* or *CHRNA3* allelic expression imbalance (AEI) in brain regions important in the reward pathway (prefrontal cortex (PFC); amygdala (Amyg) or nucleus accumbens (Nacc)) were evaluated in post-mortem brain tissue from individuals with one “risk” and one “protective” haplotype (Figure 1A) [14]. We report AEI of *CHRNA5* observed in Amyg and Nacc of one individual who was also heterozygous at distal (∼13.5 kb upstream of the transcription start site (t.s.s.)) promoter polymorphisms (rs880395 and rs7164030) as recently reported [22]. Taken together, our results serve as an independent confirmation of those of Smith *et al.* [22] and support the idea that relative risk for NA must be assessed not only based on the presence of the mis-sense cSNP rs16969968-[A] in *CHRNA5*, but also on the regulation of relative allelic expression levels.

## Results

### The CHRNA5 deletion (rs3841324-[Δ]) associates with the “mixed” European-American haplotype

Haplotype analysis indicated that the rs3841324-[Δ] allele is in strong LD (D’ = 0.965, r^2^ = 0.884) with the rs588765-[T] allele, but weaker LD (D’ = 0.961, r^2^ = 0.343) with the rs16969968-[G] allele (Figure 1B). Our analysis places rs3841324 mainly on the “second” or “mixed” haplotype (frequency of 30% in European-Americans) defined in [14] (“mixed 1” or “mixed 2” in Figure 1A). Notably, when rs3841324 is included in the haplotype analysis, we observe 3 common haplotypes (Figure 1B) similar to the observation made by Saccone *et al.* [10]; wherein joint analysis of rs588765 and rs16969968 defined 3 common haplotypes in European-Americans with different effects on risk. This implies that the cSNP at rs16969968 is not the only factor conferring risk for NA in this gene cluster.

### The “mixed” promoter haplotype confers reduced activity in vitro compared to the “risk” or “protective” promoter haplotypes

**Figure 2:**
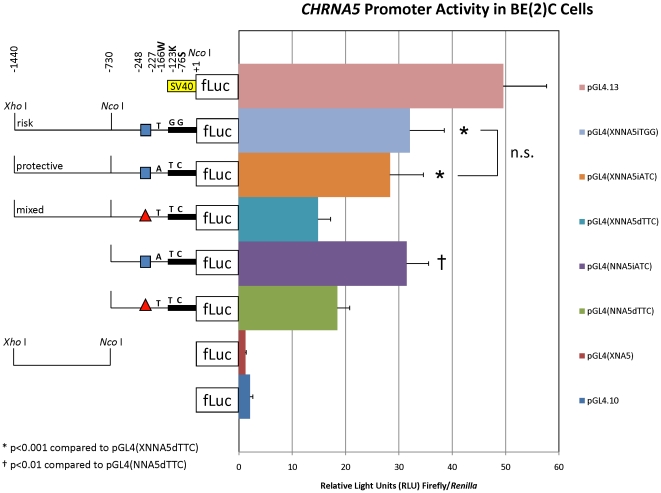
Relative *CHRNA5* promoter activity in BE(2)-C cells. Shown are the averages ± standard deviations of five separate transfections each done in triplicate. The SV40 promoter of the positive control vector pGL4.13[luc2/SV40] is indicated by a yellow box. The “promoterless” negative (background) control vector pGL4.10[luc2] is shown without a promoter region in front of the firefly luciferase (“fLuc”) reporter gene. For experimental *CHRNA5* promoter-5′UTR constructs, thin and thick black lines represent the promoter and 5′UTR portion of the construct, respectively. Positions of the *Xho* I (−1440) and *Nco* I (+1) sites used for subcloning are indicated, as is an internal *Nco* I site (−730). The positions and identities of the rs3841324 (−227 to −248), rs503464 (−166W), rs55853698 (−123K) and rs55781567 (−76S) polymorphisms within each construct are indicated. The deletion (“d” in construct name) or normal (“i” in construct name) allele at rs3841324 is indicated by a red triangle or blue square, respectively. The deletion (“d”) constructs exhibited decreased activity (1.6–2.0-fold) relative to the normal (“i”) constructs. *p<0.001 compared to pGL4(XNNA5dTTC); †p<0.01 compared to pGL4(NNA5dTTC) by ANOVA with Tukey's *post-hoc* analysis. “n.s.” indicates not significant.

Analysis of *CHRNA5* promoter haplotypes in human BE(2)-C neuroblastoma cells indicated that the “mixed” haplotype containing the rs3841324-[Δ]:rs503464-[T] variations (pGL4(XfNNA5dTTC) or pGL4(NNA5dTTC)) showed decreased (1.6–2.0-fold) relative firefly luciferase activity compared to either the “protective” haplotype containing the rs3841324-[N]:rs503464-[A] variations (*i.e.* pGL4(XNNA5iATC) or pGL4(NNA5iATC)) or the “risk” haplotype containing the rs3841324-[N]:rs503464-[T] variations (*i.e.* pGL4(XNNA5iTGG)) (Figure 2). Comparison of the analogous insertion (normal) *versus* deletion constructs showed statistically significant differences (p<0.001 and p<0.01 in the case of the longer (XNN) and shorter (NN) constructs, respectively). There was no statistically significant difference (p = 0.851) in the relative activities of the two rs3841324-[N] constructs: the “risk” haplotype (*i.e.* pGL4(XNNA5iTGG)) *versus* the “protective” haplotype (*i.e.* pGL4(XNNA5iATC)) in these cells (“n.s.” in Figure 2).

### The SP-1 transcription factor binds within the rs3841324 deletion

The rs3841324 deletion removes a few transcription factor binding sites, including SP-1 [2], [25]. Thus, we used the deleted region as probe in an SP-1-specific EMSA analysis. EMSA analysis using the sequence encompassed by the *CHRNA5* promoter deletion at rs3841324 as probe revealed specific binding of purified recombinant human SP-1 (rhSP-1) transcription factor (Figure S1). A doublet of rhSP-1 binding is present without competitor or when an AP-2 consensus sequence is used as competitor, but is absent when either cold probe or an SP-1 consensus sequence is used as competitor. This indicates that highly pure rhSP-1 binds the SP-1 site within the 22-base pair deleted region of the *CHRNA5* promoter. HeLa nuclear extract, used as a positive control for the EMSA, caused a larger mobility shift of the probe suggesting that other factors in addition to SP-1 (perhaps Erg-2 or AP-2 which also have predicted consensus binding sites within the deleted region) might interact with the *CHRNA5* promoter (Figure S1).

### The rs55853698 and rs55781567 CHRNA5 5′UTR polymorphisms affect luciferase reporter activity

**Figure 3:**
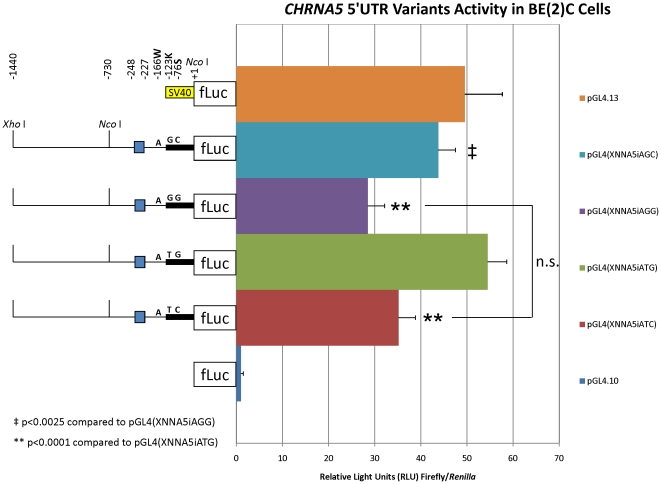
Relative *CHRNA5* 5′UTR variant luciferase activity in BE(2)-C cells. Shown are the averages ± standard deviations of four independent experiments each done in triplicate. The SV40 promoter of the positive control vector pGL4.13[luc2/SV40] is indicated by a yellow box. The “promoterless” negative (background) control vector pGL4.10[luc2] is shown without a promoter region in front of the firefly luciferase (“fLuc”) reporter gene. For experimental *CHRNA5* promoter-5′UTR constructs, thin and thick black lines represent the promoter and 5′UTR portion of the construct, respectively. Positions of the *Xho* I (−1440) and *Nco* I (+1) sites used for subcloning are indicated, as is an internal *Nco* I site (−730). The positions and identities of the rs3841324 (−227 to −248, blue box indicates the normal or “i” allele), rs503464 (−166W), rs55853698 (−123K) and rs55781567 (−76S) polymorphisms within each construct are indicated. Note that the “A” allele at rs503464 (−166 W) was not changed to “T” (as defined by haplotypes in Europeans, [26]) in the pGL(XNNA5iAGG) construct, in order to isolate the 5′UTR SNPs for effects on translation in an equivalent promoter context for each construct. ‡p<0.0025 compared to pGL4(XNNA5iAGG) and **p<0.0001 compared to pGL4(XNNA5iATG) by ANOVA with Tukey's HSD *post-hoc* analysis. “n.s.” indicates not significant.

The *CHRNA5* 5′UTR SNPs rs55853698 (T>G) and rs55781567 (C>G) do not directly impact transcription factor binding sites [7] (data not shown), although rs55853698 flanks an SP-1 binding site and rs55781567 flanks a C/EBP-β and ikaros-2 binding site. Because translation of transcribed *CHRNA5* mRNA from different promoter haplotypes (*i.e.* “risk” *versus* “protective”; see below) might be influenced by the two 5′UTR SNPs, we tested all combinations of these SNPs for effects on luciferase activity in BE(2)-C cells while keeping the promoter sequence constant. We found no statistically significant difference in luciferase activity derived from the “GG” and “TC” 5′UTR haplotypes (p = 0.46) in BE(2)C cells (Figure 3). In contrast, the “GG” 5′UTR haplotype showed statistically significant differences in luciferase activity *versus* the “GC” (p<0.0025) and “TG” (p<0.0001) 5′UTR haplotype constructs. Likewise, luciferase activity from the “TC” 5′UTR haplotype was significantly different from the “TG” haplotype (p<0.0001). Taken together, these data suggest influences of both the rs55853698 and rs55781567 SNPs in the *CHRNA5* 5′UTR on luciferase expression in BE(2)-C cells (Figure 3).

### *CHRNA3* promoter haplotypes do not affect relative transcription *in vitro*

**Figure 4:**
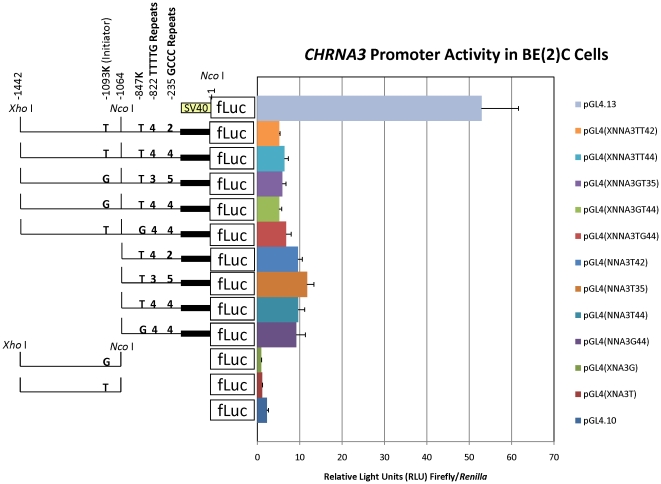
Relative *CHRNA3* promoter activity in BE(2)-C cells. Shown are the averages ± standard deviations of at least three independent experiments each done in triplicate. The SV40 promoter of the positive control vector pGL4.13[luc2/SV40] is indicated by a yellow box. The “promoterless” negative (background) control vector pGL4.10[luc2] is shown without a promoter region in front of the firefly luciferase (“fLuc”) reporter gene. For experimental *CHRNA3* promoter-5′UTR constructs, thin and thick black lines represent the promoter and 5′UTR portion of the construct, respectively. Positions of the *Xho* I (−1442) and *Nco* I (+1) sites used for subcloning are indicated, as is an internal *Nco* I site (−1064). The positions and identities of the rs13329271 (−1093 K; “initiator”) and rs12911814 (−847 K) polymorphisms, as well as the number of “TTTTG” repeats (−822 to −847) and the number of “GCCC” repeats (−235) within each construct are indicated. There were no statistically significant differences between comparisons of analogous (*i.e.* five full length, four 5′ or two 3′ truncated) promoter haplotype constructs by ANOVA with Tukey's HSD *post-hoc* analysis.

No statistically significant differences in relative firefly luciferase activity were observed among the analogous (*i.e.* pGL4(NNA3) or pGL4(XNNA3)) constructs of the *CHRNA3* promoter haplotypes tested (Figure 4).

### *CHRNA5*, but not CHRNA3, shows AEI *ex vivo*

The SNaPshot system was used to assess AEI in post-mortem brain tissue from individuals heterozygous at the *CHRNA3* and *CHRNA5* SNPs rs1051730 and rs16969968, respectively. This method is based on an expected value of 50% for gDNA amplified from heterozygotes. AEI in the mRNA-derived (cDNA) samples is indicated by deviation of either SNP allele from the expected 50% value. Because we wanted to assess AEI derived specifically from the “risk” and “protective” haplotypes, and the *CHRNA5* rs3841324-[Δ] allele associated with the “mixed” haplotype (Figure 1B) [14], [27], all individuals tested for AEI of *CHRNA5* were rs3841324-[N] homozygotes.

**Figure 5:**
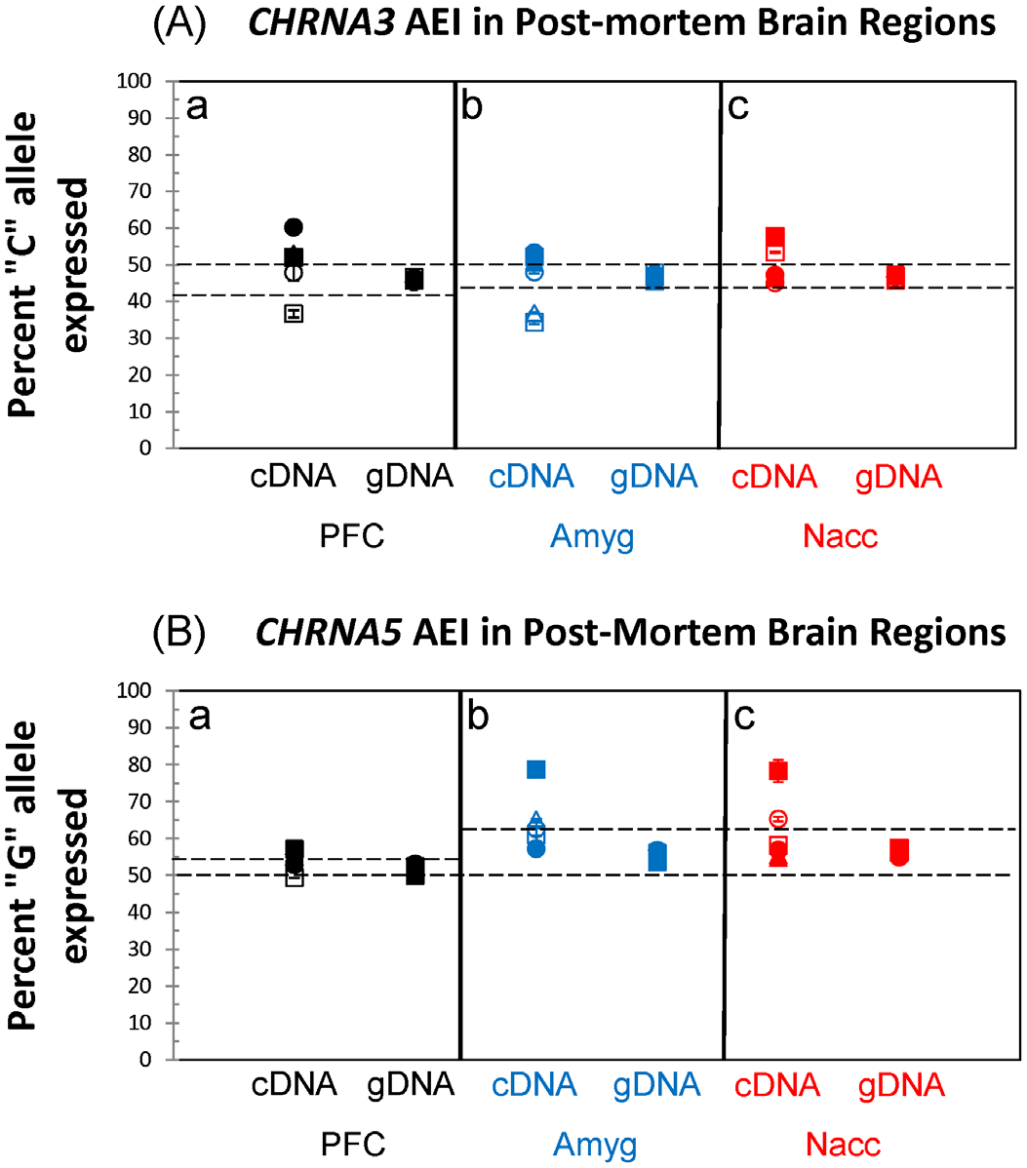
Allelic expression imbalance (AEI) of *CHRNA3* and *CHRNA5* in post-mortem brain regions. The SNaPshot system was used to assess the relative allelic expression of either *CHRNA3* (A) or *CHRNA5* (B) in complementary DNA (cDNA) preparations derived from mRNA of post-mortem brain regions, including the prefrontal cortex (PFC, black symbols), amygdala (Amyg, blue symbols) or nucleus accumbens (Nacc, red symbols). Genomic DNA (gDNA) from each individual was used as a control for no imbalance (*i.e.* 50% from either allele). The averages ± standard deviations of at least two determinations for each point are shown. (A) The percent rs1051730-[C] (%C) allelic expression from *CHRNA3* is shown for all heterozygous individuals tested in the PFC (black symbols; individuals 1 through 6 are represented as closed squares, open squares, closed triangles, open triangles, closed circles or open circles, respectively), the Amyg (blue symbols; individuals 7 through 12 are represented as closed squares, open squares, closed triangles, open triangles, closed circles or open circles, respectively) or the Nacc (red symbols; individuals 7 through 12 are represented as closed squares, open squares, closed triangles, open triangles, closed circles or open circles, respectively). Dashed lines represent the degree of error in our standard curve method as determined by the formula ((expected %C in gDNA-observed %C in gDNA)/expected %C in gDNA). This corresponds to 8% in the PFC determinations (panel “a”) and 7% error in the Amyg (panel “b”) and Nacc (panel “c”) determinations. (B) The percent rs16969968-[G] (%G) allelic expression from *CHRNA5* is shown for all heterozygous individuals tested in the PFC (black symbols; individuals 1 through 6 are represented as closed squares, open squares, closed triangles, open triangles, closed circles or open circles, respectively), the Amyg (blue symbols; individuals 7, 8, and 10 through 12 are represented as closed squares, open squares, and open triangles, closed circles or open circles, respectively) or the Nacc (red symbols; individuals 7 through 12 are represented as closed squares, open squares, closed triangles, open triangles, closed circles or open circles, respectively). Dashed lines represent the degree of error in our standard curve method as determined by the formula ((expected %G in gDNA-observed %G in gDNA)/expected %G in gDNA). This corresponds to 3% in the PFC determinations (panel “a”) and 11% error in the Amyg (panel “b”) and Nacc (panel “c”) determinations. Note individual “7” (closed square) showing 80% rs16966968-[G] expression in both the Amyg (blue square, panel “b”) and Nacc (red square, panel “c”).

Analysis of individual sample means (cDNA *versus* gDNA) indicated statistically significant (p<0.05; ANOVA with Tukey-Kramer HSD *post-hoc*) AEI for both *CHRNA3* and *CHRNA5* (Figure 5); however, when all determinations for a particular brain region were grouped, there was no statistical evidence of AEI for *CHRNA3* (PFC: Levene's test for equal variance, p = 0.0382; Welch's test for equal means, p = 0.2189; Amyg: Levene's, p = 0.0012; Welch's, p = 0.8486; Nacc: Levene's, p<0.0001; Welch's, p = 0.1022). Likewise, there was no statistical evidence for AEI in grouped determinations of *CHRNA5* in PFC (Levene's, p = 0.0839; Welch's, p = 0.0606). In contrast, for *CHRNA5*, grouped determinations for Amyg and Nacc revealed statistically significance differences in allelic expression (Amyg: Levene's, p = 0.0232, Welch's, p = 0.0025; Nacc: Levene's, p = 0.0006; Welch's, p = 0.050). In both cases, individual “7” (∼80%G expressed) appears to be driving the statistical result (Figure 5B) because statistical significance is no longer observed (*i.e.* p>0.05) when this individual is removed from the analysis. Whereas the smoking history of individual “7” could not be determined, we note that neither diagnosis (normal, schizophrenic or Alzheimer's) nor smoking history of an individual was concordant with whether an individual showed AEI. Whereas the 80:20 AEI between *CHRNA5* alleles in both the Nacc and Amyg of individual “7” is probably biologically significant (Figure 5B), the biological relevance of the “suggestive” AEIs for *CHRNA3* and *CHRNA5* in individuals other than “7” remains unclear as they were observed in only one brain region, were in opposite directions in different brain regions from the same individual or were small enough to be within the precision limits of the assays (Figure 5). Indeed, *CHRNA3* promoter haplotypes for those individuals studied were not in concordance with any suggestive AEI of *CHRNA3* (Figure 5A and Table S2). Importantly, both methods used for determining AEI in each sample (standard curve *versus* formula of [28]) gave similar results, with individual “7” showing *CHRNA5* AEI (Figures S2A and S2B).

### *CHRNA5* AEI is concordant with heterozygosity at 5′ distal promoter SNPs, but not with an intronic SNP

The intronic rs588765-[T] allele correlates with increased steady-state *CHRNA5* mRNA levels in post-mortem PFC of rs3841324-[Δ] homozygotes [27] and rs588765-[T] is in strong LD (r^2^ = 0.88) with the rs3841324-[Δ] background in people of European ancestry (Figure 1B) [10]. Because we studied AEI only in rs3841324-[N] homozygotes, the observed 80:20 *CHRNA5* imbalance might have been explained by recombination which placed the rs588765-[T] allele on the rs3841324-[N]:rs16969968-[G] “protective” background; this possibility is supported by haplotype analysis showing ∼2% of Europeans have such an association of SNPs (“rare” in Figure 1B). However, because individual “7” was homozygous for the “C” allele at rs588765 (data not shown), such recombination could not explain this observation. Further, sequencing of amplified gDNA from this individual's *CHRNA5* core promoter (which was heterozygous for the “W” SNP (rs503464) at −166, the “K” SNP (rs55853698) at −123 and the “S” SNP (rs55781567) at −76; data not shown) showed no evidence of a rare variant that might alter a binding site for transcription factors. A recent report found a positive correlation between *CHRNA5* AEI in PFC and the minor alleles of three SNPs (rs880395, rs905740 and rs7164030) in the extremely 5′ distal *CHRNA5* promoter region (∼13.5 kb 5′ of the t.s.s.) [22]. We amplified this region of *CHRNA5* from gDNA of all individuals tested for AEI and genotyped two of the three SNPs (rs880395 and rs7164030) by RFLP analysis. Whereas individual “7”, who showed convincing AEI of *CHRNA5* (Figure 5B), was heterozygous at both rs880395 and rs7164030 (Figure S3), all other individuals in our study, who showed no statistically significant *CHRNA5* AEI (Figure 5B and Figure S2B), were homozygous for the major alleles at these SNPs (Figure S3). These results serve as an independent confirmation of those reported previously [22].

### The 5′ distal *CHRNA5* promoter region acts as a strong transcriptional repressor

**Figure 6:**
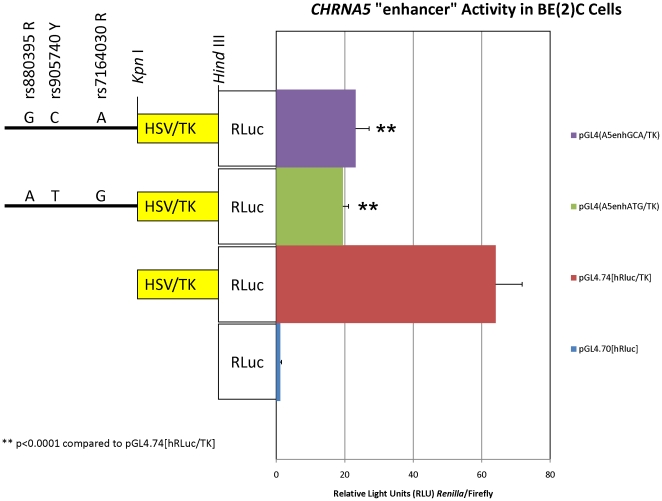
5′ distal *CHRNA5* promoter element acts as transcriptional repressor. Two haplotypes of an 852 bp portion of the 5′ distal region of the *CHRNA5* promoter (13.5 kb upstream of the t.s.s.) was sub-cloned upstream of the HSV-TK promoter and transfected into BE(2)C cells. Shown are the averages ± standard deviations of four independent experiments each done in triplicate. The HSV-TK promoter of the positive control vector pGL4.74[hRluc/TK] and experimental heterologous promoter constructs is indicated by a yellow box. The “promoterless” negative (background) control vector pGL4.70[hRluc] is shown without a promoter region in front of the *Renilla* luciferase (“RLuc”) reporter gene. The 5′ distal region of the *CHRNA5* promoter is indicated by a solid black line. The positions and identities of the rs880395 (R), rs905740 (Y) and rs7164030 (R) SNPs within each construct are indicated. Whereas the 5′ distal element acted as a strong repressor (**p<0.0001 compared to pGL4.74[hRluc/TK]), there were no statistically significant differences between comparisons of the two promoter haplotype constructs (p = 0.183) by ANOVA with Tukey's HSD *post-hoc* analysis.

To investigate the function (enhancer or repressor) of the distal promoter region containing the three SNPs (rs880395, rs905740 and rs7164030) associated with AEI [22], we amplified the “risk” or “protective” (*i.e.* GCA) and the “mixed” (*i.e.* ATG) haplotypes of the region (Figure 1A) and created heterologous promoter constructs. We found that this 5′ distal promoter region acts as a strong repressor of transcription when joined upstream of the HSV-TK promoter (Figure 6). Importantly, and somewhat unexpectedly, we observed no statistically significant difference in relative promoter activity from the two haplotype constructs (p = 0.183) in BE(2)C cells (Figure 6).

## Discussion

Both genome-wide and candidate gene association studies have pointed to the *CHRNA5*-*CHRNA3*-*CHRNB4* cluster of nAChR subunit genes as being important in the etiology of NA, lung cancer, peripheral artery disease and chronic obstructive pulmonary disease [7]–[18], [29]. With respect to the *CHRNA5-A3-B4* gene cluster in people of European descent, 4 common and 2 rare haplotypes have been defined [14] (shown in Figure 1A; with an additional rare “mixed 2” haplotype). Notably, *CHRNA5* and *CHRNA3* constitute a single haplotype block making the assignment of functionally causative alleles for these various phenotypes difficult.

We observed no statistically significant differences in relative *CHRNA3* promoter activity among haplotypes studied in human neuroblastoma BE(2)-C cells (Figure 4) and no statistically significant AEI of *CHRNA3* mRNA in the brain regions examined (Figure 5A and Figure S2A). Taken together, these data suggest that overall relative risk for the development of NA among heterozygotes for the “risk” and “protective” haplotypes does not depend on *CHRNA3* AEI due to the promoter and/or cSNPs (within the mRNA) that comprise each examined haplotype. Further, no concordance between *CHRNA3* promoter haplotypes and any “suggestive” AEI was observed (Table S2). One caveat to these studies is the observation that *CHRNA3* expression is regulated by promoter methylation [30]. Thus, we cannot exclude differential promoter methylation within different brain regions for those samples that showed “suggestive” AEI of *CHRNA3*.

In contrast to *CHRNA3*, *CHRNA5* promoter haplotypes showed statistically significant differences in relative promoter activity when compared in BE(2)-C cells; however, these statistical differences were not between the “risk” and “protective” promoter haplotypes, but rather between the “risk” or “protective” haplotype and the “mixed” promoter haplotype containing the deletion at rs3841324 (Figure 2). The rs3841324-[Δ] allele has no effect on transcription in TE671 cells [25], but decreases transcription by ∼40% in HEK293T cells [25]. Similarly, in three of four lung cancer cell lines examined, rs3841324-[Δ] caused an ∼80% decrease in *CHRNA5* promoter-derived luciferase activity [26]. In contrast to these *in vitro* data, *ex vivo* data from post-mortem brain (PFC) and resected lung tissue indicated that *CHRNA5* rs3841324-[Δ] homozygotes generate ∼2.5-3-fold higher *CHRNA5* steady-state mRNA levels than *CHRNA5* rs3841324-[N] homozygotes [26], [27]. Thus, whereas our data in BE(2)-C cells agree with most of these *in vitro* studies, indicating decreased relative *CHRNA5* promoter activity from the promoter with a deletion at rs3841324, the e*x vivo* studies suggest the opposite effect [26], [27].

The rs3841324 deletion encompasses a few transcription factor binding sites, including SP-1 [2], [25], a transcription factor which activates transcription. The large shift of the probe with HeLa nuclear extract might indicate binding of factors other than SP-1 because the mobility shift is greater than with rhSP-1 alone (Figure S1). Nonetheless, although we cannot exclude the impact of other factors, such as Erg-2 and AP-2, which might regulate brain expression of *CHRNA5* by binding within the promoter deletion at rs3841324, our EMSA data indicate that pure rhSP-1 binds within this deleted region (Figure S1). Given that SP-1 stimulates transcription, the loss of a binding site for SP-1 caused by the rs3841324 deletion could explain, in part, the decreased *in vitro* transcriptional activity from the *CHRNA5* rs3841324-[Δ] construct in most cell types, but not the enhanced activity observed from this allele *ex vivo* [26], [27].

In the study of post-mortem brain [27], the higher *CHRNA5* steady-state mRNA levels from the rs3841324-[Δ] allele were associated with the rs588765-[T] allele in intron 1 of *CHRNA5*. Furthermore, *CHRNA5* distal promoter (∼13.5 kb upstream of the t.s.s.) polymorphisms correlated with the levels of *CHRNA5* mRNA alleles expressed *ex vivo* [22], a finding that we confirmed in our limited *CHRNA5* AEI experiments (Figure 5B and Figure S2B). Thus, an alternative and simple explanation for the contradictory (*in vitro versus ex vivo*) findings may be the exclusion of regions of the *CHRNA5* gene (distal promoter or intron 1 where rs588765 is located) from the constructs used in the *in vitro* transfection studies that are necessary for determining the relative allelic transcriptional activity *in vivo*.

Because the rs3841324-[Δ] allele is in “substantial LD” with the minor alleles (“ATG”) of the distal promoter SNPs [22] and is also in strong LD with the “mixed” European-American haplotype (Figure 1A), it was hypothesized that transcription factors binding putative “enhancer” elements within the distal portion of the *CHRNA5* promoter might increase expression of the rs16969968-[G] allele from the “mixed” haplotype *in vivo* [22]. We restricted our analysis of *CHRNA5* AEI to individuals with two rs3841324-[N] alleles at the *CHRNA5* promoter. As such, we assessed the effect of all SNPs within the “risk” or “protective” haplotypes on the relative allelic mRNA levels of *CHRNA5* by interrogating heterozygotes for these two haplotypes. As with *CHRNA3*, most individuals showed no AEI in steady-state *CHRNA5* mRNA in the post-mortem brain regions examined (Figure 5B). However, one individual (“7”) showed an 80:20 %G:%A ratio of *CHRNA5* steady-state mRNA in favor of the “protective” rs16969968-[G] allele (Figure 5B). Neither recombination between the rs3841324-[N] allele and the intronic rs588765-[T] allele, which was found to be associated with higher steady-state mRNA levels in PFC [27], nor a rare variant in this individual's *CHRNA5* core promoter explained this finding. However, we found heterozygosity at rs880395 and rs7164030 (Figure S3) in this individual's distal *CHRNA5* promoter that is concordant with the observed AEI (Figure 5B) and with correlative findings in other studies [22]. Notably, in contrast to the “mixed” haplotype where the minor alleles (“ATG”) are associated, haplotype analysis indicates that the major alleles (“GCA”) at these distal promoter SNPs most often associate with the “risk” and “protective” haplotypes (Figure 1A).

The individual who showed AEI of *CHRNA5* (“7”; Figure 5B) expressed ∼4-fold more of the rs16969968-[G] “protective” allele relative to the rs16969968-[A] “risk” allele. Furthermore, out of those examined (n = 6 for PFC and n = 6 for Amyg and Nacc), this individual was the only one to show clear AEI and to have the minor alleles (“ATG”) at the 5′ distal promoter SNPs rs880395 and rs7164030 (Figure S3). Given the association of the major alleles (“GCA”) at these distal promoter SNPs with mainly the “risk” and “protective” haplotypes (Figure 1A), we hypothesize that this individual (“7”) might be a recombinant with the minor alleles (“ATG”) now associated with the “protective” haplotype. If the minor alleles of these distal promoter SNPs fall on the normal (non-deleted) rs3841324 “protective” haplotype (as might be the case in individual “7”), the distal promoter SNPs might drive 4-fold greater *CHRNA5* expression as compared to ∼2.5–3-fold greater expression when the deletion is present at rs3841324 on the “mixed” haplotype. Thus, if the cSNP rs16969968 in *CHRNA5* confers risk for NA, in a heterozygote (“risk” (rs16969968-[A]) *versus* “protective” or “mixed” (rs16969968-[G]) haplotype), the ‘comprehensive’ relative risk would depend on the genotypes at rs16969968, rs3841324 and these distal promoter SNPs.

When we tested directly the two haplotypes of this 5′ distal *CHRNA5* promoter region, however, we found that it acts as a strong repressor, rather than an enhancer, of transcription from the HSV-TK promoter (Figure 6). Quite unexpectedly, there were no statistically significant differences between the promoter activities of the two haplotype constructs (Figure 6). We cannot exclude the possibilities that elements within this 5′ distal region of *CHRNA5* might act differently when placed at a greater distance from the transcription start site (normally ∼13.5 kb upstream of the t.s.s.) or when linked to the native *CHRNA5* promoter which is not a TATA-containing promoter [2] as is the HSV-TK promoter. Nonetheless, taken together with the finding that the “risk” and “protective” promoter haplotypes showed no statistically significant difference in promoter activity (Figure 2), the observation of 80:20 AEI of *CHRNA5* in individual “7” (Figure 5B) might also suggest an expression effect of SNPs within the coding, other than rs16969968, or untranslated regions of the *CHRNA5* transcript.

A recent meta-analysis of NA genome-wide association studies revealed a strong association signal from rs55853698 in the *CHRNA5* 5′UTR [7]. We analyzed combinations of the *CHRNA5* 5′UTR SNPs rs55853698 and rs55781567, on the rs3841324-[N]:rs503464-[A] *CHRNA5* promoter background, in BE(2)-C cells. Whereas neither rs55853698 nor rs55781567 is predicted to alter a transcription factor binding site [7], both flank predicted transcription factor binding sites (see Results above). There was no statistically significant difference in activity observed from the “risk” “GG” *versus* “protective” “TC” haplotype constructs (Figure 3). Interestingly, the “risk” “GG” construct showed a statistically significant difference in activity compared to both the “TG” and “GC” constructs (Figure 3) suggesting an influence of rs55853698 and rs55781567, respectively. Thus, these data suggest a possible role for 5′UTR SNPs in translational regulation of firefly luciferase in BE(2)-C cells, and perhaps a translational regulatory component to *CHRNA5* expression. Given the occurrence of the “GG” or “TC” diplotypes on the “risk” or “protective” and “mixed” European haplotypes, respectively, the biological significance of this effect remains unclear. We conclude that, in addition to rs16969968, overall risk for NA in populations of European ancestry must be assessed based on the contributions of other SNPs within the extended haplotype of *CHRNA5* which may influence expression levels of *CHRNA5* mRNA, and perhaps also α5 protein.

## Materials and Methods

### Subjects

Post-mortem brain samples were obtained from either the University of Pennsylvania Brain Bank (n = 6; PFC) or the Golden Brain Bank at the Department of Veterans Affairs (VA) Medical Center - Coatesville, PA (n = 6; Amyg and Nacc). For both brain banks, subjects were excluded for prolonged agonal state (unresponsive>24 hrs), ventilator support (>12 hrs), and history of major, known CNS disease apart from the disease recorded (normal, schizophrenia or Alzheimer's). Patients for whom the post-mortem interval was>24 hrs were excluded. Patients were excluded on the basis of i) alternate or ambiguous DSM-IV diagnosis, ii) substance abuse (of illicit substances, but not ethanol or nicotine), iii) neurological disorder predating the onset of psychiatric symptoms (*e.g.*, epilepsy, traumatic brain injury, etc.), iv) subsequent neurological disorders that would significantly compromise brain function (*e.g.*, anoxia, stroke) and confound interpretation of postmortem findings. All subjects were of European ancestry as determined at time of autopsy. All samples were obtained with approval from the respective institutional review boards (by an IRB at the University of Pennsylvania or by the Veterans Integrated Service Network 4 Multi-Site IRB at Coatesville VAMC) and following all brain bank user agreements.

### Haplotype analysis of *CHRNA5* and *CHRNA3* SNPs in the European-American population

Genomic DNA (gDNA) from individuals of European ancestry from the University of Pennsylvania Brain Bank (n = 80) were genotyped for six polymorphisms across the *CHRNA5* and *CHRNA3* genes. For *CHRNA5*, the normal ([N]) *versus* 22 base pair deletion ([Δ]) allele at rs3841324 in the *CHRNA5* core promoter was genotyped by PCR using primers (see Table S1 for all PCR primers and parameters used) that flanked the deletion site. Alleles were separated on either a 5% non-denaturing polyacrylamide or a 2.5% agarose gel system. For SNPs rs588765, rs637137, and rs16969968 in *CHRNA5* and SNPs rs1051730 and rs8040868 in *CHRNA3*, genotypes were determined using TaqMan® SNP genotyping assays C_18826_10, C_5866_10, C_26000428_20, C_9510307_20 and C_261698_10; respectively (Life Technologies, Carlsbad, CA) in a standard 5 µl PCR using an Applied Biosystems, Inc. (ABI) 384-well block 9700 under standard cycling parameters. Plates were post-read on an ABI 7900HT using the SDS 2.2.2 software. All calls were entered into an Excel spreadsheet prior to analysis of LD and haplotype structures using Haploview 4.2 software [23].

### Functional Analysis of *CHRNA5* and *CHRNA3* promoter-5′UTR haplotypes in BE(2)-C cells

Human BE(2)-C (CRL-2268) neuroblastoma cells (a sub line of human SK-N-BE(2) cells which express both *CHRNA5* and *CHRNA3* (GAD, unpublished data)) were obtained from the American Type Culture Collection (ATCC; Manassas, VA) and propagated in a 37°C humidified incubation chamber with 5% CO_2_ in a 1:1 mixture of Eagle's Minimal Essential Medium (ATCC):Ham's F12 (Life Technologies) containing 10% fetal bovine serum (Thermo Scientific [Hyclone]; Rockford, IL). The *CHRNA5* core promoter and 5′UTR (-1440 to +1 at the AUG start codon) was amplified from gDNA of an individual heterozygous at rs3841324, but homozygous G/G at rs16969968, in an initial PCR using forward and reverse primers that generated 1798 bp amplicons, and that yielded the “mixed” and “protective” *CHRNA5* promoter haplotypes (see Figure 1A). For *CHRNA3*, the core promoter and 5′UTR (-1442 to +1 at the AUG start codon) was amplified from gDNA of different individuals in an initial PCR using forward and reverse primers that generated 1535 bp amplicons. Resulting *CHRNA5* and *CHRNA3* promoter amplicons were subcloned by T/A-cloning into pCRII-TOPO using the Dual Promoter T/A-TOPO-cloning kit (Invitrogen, Carlsbad, CA). Plasmid DNA from various clones was prepared by mini-preparation using the GeneElute Miniprep system (Sigma, Saint Louis, MO). Clones without mutations (determined by Sanger sequencing of each clone) were diluted to 1 ng/µl before use in a second “half-nested” PCR employing the same forward primers (which both had *Xho* I sites incorporated at their 5′ ends) as in the initial PCR, but a new reverse primer with an incorporated *Nco* I site to facilitate subsequent subcloning (see below). We note that the *Nco* I site changes a “G” nucleotide to a “C” nucleotide at the -1 position in the *CHRNA5* promoter. Resulting amplicons (from the second PCR) were cloned into pCRII-TOPO and plasmid DNA from various clones was prepared by mini-preparation and checked for unwanted mutations as above. pCRII clones without mutations were then digested with *Xho* I and *Nco* I prior to gel purification and subsequent sub-cloning of the promoters into *Xho* I/*Nco* I-cut pGL4.10[*luc*2] (Promega, Madison, WI, USA) using T4 DNA ligase (New England Biolabs, Beverly, MA). We performed three QuikChange® (Agilent) site-directed mutagenesis reactions (see Table S1 for all QuikChange® primers and PCR parameters) to generate the “risk” *CHRNA5* promoter from the “protective” *CHRNA5* promoter construct (*i.e.* pGL4(XXNA5iATC); see above). Prior to use in transfections, each construct was prepared by maxi-preparation using Purelink columns (Life Technologies) and the promoter region fully sequenced to check for unwanted mutations. Experimental firefly luciferase promoter constructs (1.96 µg) and pGL4.74[hRLuc/TK] (Promega) vector (0.04 µg) were transiently co-transfected into human BE(2)-C neuroblastoma cells using Trans-it LT1 reagent (Mirus, Madison, WI). After 48 hrs, firefly and *Renilla* luciferase expression levels were assessed using a “Dual-Ready” TD-20/20 luminometer (Turner Biosystems, Sunnyvale, CA). Firefly luciferase activity (in light units [LU]) was normalized by dividing by *Renilla* luciferase activity (in LUs). For each experimental series, duplicate or triplicate transfections were done and the normalized (firefly/*Renilla*) values (in relative LUs) were averaged and counted as one determination. At least five (*CHRNA5*) or three (*CHRNA3*) separate experimental series were conducted and determinations were averaged, graphed and used in statistical analysis.

To test if the *CHRNA5* 5′UTR SNPs rs55853698 and rs55781567 might influence translation (of firefly luciferase), the original “protective” promoter construct (*i.e.* pGL4(XNNA5iATC); see above) was mutagenized to all four possible 5′UTR 2-SNP combinations using the QuikChange® site-directed mutagenesis kit (Agilent). Mutagenized plasmids were prepared by maxi-preparation and promoter regions sequenced in entirety to check for undesired (PCR-based) mutations before being transfected into human BE(2)-C neuroblastoma cells for analysis as described above. For each experimental series, triplicate transfections were done and the normalized (firefly/*Renilla*) values (in relative LUs) were averaged and counted as one determination. Four separate experimental series were done and the 4 determinations were then averaged, graphed and used for statistical analysis.

To test if the distal promoter region of *CHRNA5*, containing SNPs rs880395, rs905740 and rs7164030 (Figure 1A), could function as an enhancer, we amplified an 852 bp region of the distal *CHRNA5* promoter (∼13.5 kb 5′ of the t.s.s.) from gDNA of individual #7 (Figure 5) who was heterozygous at rs880395 and rs7164030 (Figure S3) by PCR with forward and reverse primers (Table S1) using the SequalPrep Long Distance PCR Kit (Life Technologies). PCR amplicons were then sub-cloned into the pCRII-TOPO vector using the Dual Promoter T/A-TOPO cloning kit (Life Technologies). Plasmid DNA from positive clones was prepared by mini-preparation using the GeneElute system (Sigma) and was fully sequenced to determine the orientation and identity of the putative enhancer haplotype sequence. Two haplotypes of rs880395, rs905740 and rs7164030 were identified: A-T-G and G-C-A, respectively. pCRII plasmid DNA of clones with the distal promoter haplotypes in the “negative” orientation were digested with *Kpn* I and *Hind* III restriction enzymes and the Herpes simplex virus thymidine kinase promoter (HSV-TK) fragment (removed from pGL4.74[hRLuc/TK] (Promega) by digestion with *Kpn* I and *Hind* III) was sub-cloned into the above pCRII vector using T4 DNA ligase (New England Biolabs). The resulting constructs were then digested with *Eco* RV and *Hind* III, the *CHRNA5* “A5 enhancer”-HSV-TK fragment gel purified and then ligated into pGL4.74[hRLuc/TK] (Promega) that had been digested with *Acc* 65I, blunted by a fill-in reaction with Klenow fragment (NEB) and then digested with *Hind* III. This resulted in the creation of plasmids pGL4(A5enhATG/TK) and pGL4(A5enhGCA/TK) which were prepared by maxi-preparation, fully sequenced and then used in transfection experiments. Because *Renilla* luciferase was the reporter for these assays, the pGL4.13[luc2/SV40] (Promega) vector (which expresses firefly luciferase from the SV40 promoter) was co-transfected and used to normalize the *Renilla* luciferase expression from the experimental plasmids. Briefly, experimental *Renilla* luciferase promoter constructs (1.96 µg) and pGL4.13[luc2/SV40] (Promega) vector (0.04 µg) were transiently co-transfected into human BE(2)-C neuroblastoma cells using Trans-it LT1 reagent (Mirus). After 48 hrs, firefly and *Renilla* luciferase expression levels were assessed using a “Dual-Ready” TD-20/20 luminometer (Turner Biosystems). *Renilla* luciferase activity (in light units [LU]) was normalized by dividing by firefly luciferase activity (in LUs). For this experimental series, duplicate or triplicate transfections were done (total of 5 replicate determinations for each experimental construct) and the normalized (*Renilla*/firefly) values (in relative LUs) were averaged and counted as one determination. Five separate experimental series were done and the 10 determinations were then averaged, graphed and used for statistical analysis.

### Electrophoretic mobility shift assays (EMSA)

Because an SP-1 site is located within the DNA sequence encompassing the *CHRNA5* promoter deletion at rs3841324, we used the deleted region of the *CHRNA5* promoter as probe in an SP-1-specific EMSA analysis. Briefly, 100 pmol each of TOP (5′-GATTGGGCGGGGCCAGAGGGAAATAGGGG-3′) and BOTTOM (5′-CCCCTATTTCCCTCTGGCCCCGCCCAATC-3′) oligomers were made double-stranded by heating to 75°C for 10 min in 1X oligomer annealing buffer (10 mM Tris-HCl; pH 7.5, 1 mM EDTA, 100 mM NaCl), then slow-cooled in 10°C steps of 65°C for 5 min, 55°C, 45°C, and 35°C, each for 30 sec, then held at 25°C until further use. Double-stranded DNA oligomers (100 pmol) were radio-labeled in the presence of γ-[^32^P]-ATP using T4 polynucleotide kinase under standard conditions. The reaction was stopped by addition of 1 µl of 0.5 M EDTA and 89 µl TE (10 mM Tris-HCl; pH 7.6, 1 mM EDTA) prior to purification through a G-25 Sephadex spin column (Roche, Nutley, NJ). EMSA was performed essentially as outlined in the Gel Shift Assay System (Promega) instructions. Briefly, 300 ng of recombinant human (rh)SP-1 (Promega) were incubated for 10 min at 25°C in the absence or presence of 50 or 100 molar excess unlabeled double-stranded probe, specific (SP-1) or non-specific (AP-2) binding site oligomers prior to addition of 125 fmol radio-labeled double-stranded probe. As a positive control, 10 µg of HeLa cell nuclear extract was used in the gel shift assay. After addition of radio-labeled probe, proteins (HeLa or rhSP-1) were allowed to bind for 20 min at 25°C. Reaction products were separated on a 4% non-denaturing polyacrylamide gel, the gel dried and exposed to a Phosphor screen. Bands were visualized using a Typhoon Phosphor imager (GE Healthcare, Piscataway, NJ).

### Assessment of *CHRNA5* and *CHRNA3* AEI in post-mortem brain

The SNaPshot (Life Technologies) SNP genotyping system was used to determine the specific levels of “risk” or “protective” alleles expressed from *CHRNA5* (at 16969968) or from *CHRNA3* (at rs1051730). SNaPshot has been adapted successfully by a number of investigators for the purpose of determining AEI [22], [28], [31]–[34]. Because the SNaPshot system relies on *post-hoc* analysis of PCR amplicons of cDNA, care was taken to design primers for the amplification step that were within regions without known SNPs. Furthermore, we determined the cycle thresholds for *CHRNA5* (Hs00181248_m1, ABI) and *CHRNA3* (Hs00609519_m1, ABI) using quantitative RT-PCR gene expression assays (which do not distinguish relative allelic contributions to steady-state mRNA levels) on cDNA libraries created from post-mortem brain mRNA. From these qRT-PCR values, care was then taken to ensure that we were in the linear range of PCR amplifications for the AEI assays using SNaPshot. Total RNA was isolated from post-mortem brain tissue regions of individuals (PFC, n = 6; Amyg or Nacc, n = 6) heterozygous (*i.e.* with one “risk” and one “protective” allele) at rs16969968 and rs1051730 using the Trizol® reagent (Life Technologies) method. Prior to reverse transcription, 2-5 µg of total RNA was treated with *DNAse* I (Sigma-Aldrich, Saint Louis, MO) for 15–30 mins at 25°C. *DNAse* I digestion was stopped by adding EDTA, heating reactions to 70°C for 10 mins and quenching on ice before reverse transcription with random hexamers and SuperScript II® reverse transcriptase using the First-strand cDNA synthesis kit (Life Technologies). First strand cDNA was treated with RNase H at 37°C for 20 min before use in exon-specific PCR which amplified portions of exon 5 from *CHRNA5* or *CHRNA3* cDNAs containing rs16969968 and rs1051730, respectively. For *CHRNA5* or *CHRNA3*, PCR primers (Table S1) amplified a 198 bp or 160 bp amplicon, respectively, that was then used in SNaPshot analysis.

Standard curves for SNaPshot analysis were created by analyzing mixtures of exon 5 amplicons from gDNA from individuals homozygous at rs1051730 (CC or TT) or at rs16969968 (GG or AA) combined in multiple CC:TT or GG:AA ratios (100:0, 90:10, 80:20, 70:30, 60:40, 50:50, 40:60, 30:70, 20:80, 10:90 and 0∶100). To determine AEI, the exon 5 region of *CHRNA5* or *CHRNA3* was amplified from cDNA and gDNA from the same (heterozygous) individual and then PCR products were analyzed using the SNaPshot Multiplex Kit (Life Technologies). Briefly, 0.01 to 0.04 pmol of diluted gDNA and cDNA PCR products were thermocycled for 25 cycles in duplicate singleplex reactions in the presence of the appropriate PAGE-purified primer: (5′-CTAGAAACACATTGGAAGCTGCGCTC-3′ for rs16969968; 5′- ATCAAAGCCCCAGGCTA-3′ for rs1051730) and fluorescently-labeled dideoxyNTPs. Reaction products were analyzed on an ABI 3730 bioanalyzer using a POP7 matrix and LIZ 120 standards. Peak areas were calculated with Peak Scanner 1.0 software (Life Technologies). The ratio of the peak areas of each SNP was taken from the mixed homozygous allele reactions and used to generate a standard curve in Excel. For experimental (cDNA) and control (gDNA) reactions from heterozygous individuals, the ratio of each peak area was calculated and then the percent “G” (rs16969968) or percent “C” (rs1051730) allele was determined using the standard curve for each respective SNP. To verify that our method of AEI determination was accurate, we also quantified each sample using the formula: 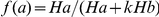
where f_(a)_ is the relative frequency of the ‘a’ allele expression, H_a_ and H_b_ are the peak heights of alleles ‘a’ and ‘b’ in cDNA and *k* corrects for the unequal amplification of SNP alleles in a heterozygous gDNA sample and is equal to ‘a/b’ where ‘a’ and ‘b’ are the peak heights of the two alleles in gDNA [28].

### Restriction fragment length polymorphism (RFLP) analysis of the *CHRNA5* upstream promoter region

An 852 bp region of the distal *CHRNA5* promoter (∼13.5 kb 5′ of the t.s.s.) was amplified by PCR. For RFLP analysis, each amplicon was digested with either *Alu* I (rs880395) or *Dde* I (rs7164030). Digestion products were resolved on a 5% non-denaturing polyacrylamide gel, post-stained with ethidium bromide and visualized and documented under UV trans-illumination.

### Statistical Analysis

Relative promoter activities were compared by multi-way ANOVA followed by Tukey's “Honestly Significant Difference” (HSD) *post-hoc* analysis. AEI in specific brain regions was assessed statistically by Welch's ANOVA because of suspected heterogeneous variance, which was confirmed for most groups by Levene's test. JMP 8.0 software was used for all statistical analyses and a *p*-value<0.05 was considered statistically significant in all tests.

## Supporting Information

Figure S1 ***CHRNA5*** **promoter InDel (rs3841324) sequence binds SP1.** Radio-labeled double-stranded oligomers encompassing the *CHRNA5* promoter deletion (rs3841324, −227 to −248) were used as probe for binding to 300 ng of pure recombinant human SP-1 (rhSP-1) protein or 10 µg of HeLa nuclear extract. HeLa and rhSP-1 specific bands are indicated by double gray and double black arrows, respectively. The single black arrow indicates the radio-labeled free probe (“dsA5pInDel*”). Right triangles at the top of the schematic indicate lanes with increasing molar excess (50X or 100X) of competitors. Probe alone (lane 1); probe with HeLa nuclear extract only (lane 2); probe with rhSP-1 only (lane 3); probe with rhSP1 and 50 or 100 molar excess of cold probe as competitor (lanes 4 and 5, respectively); probe with rhSP-1 and 50 or 100 molar excess of cold SP-1 consensus oligomer as specific competitor (lanes 6 and 7, respectively); probe with rhSP1 and 50 or 100 molar excess of cold AP-2 consensus oligomer (which, like the SP-1 site, is GC-rich) as non-specific competitor (lanes 8 and 9, respectively).
(TIF)

Figure S2
**Allelic expression imbalance (AEI) of** ***CHRNA3*** **and** ***CHRNA5*** **in post-mortem brain regions.** The SNaPshot system was used to assess the relative allelic expression of either *CHRNA3* (A) or *CHRNA5* (B) in complementary DNA (cDNA) preparations derived from mRNA of post-mortem brain regions, including the prefrontal cortex (PFC, black symbols), amygdala (Amyg, blue symbols) or nucleus accumbens (Nacc, red symbols). Allelic expression was quantified for each sample using the formula: 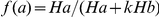 where f_(a)_ is the relative frequency of the ‘a’ allele expression, H_a_ and H_b_ are the peak heights of alleles ‘a’ and ‘b’ in cDNA and *k* corrects for the unequal amplification of SNP alleles in a heterozygous gDNA sample and is equal to ‘a/b’ where ‘a’ and ‘b’ are the peak heights of the two alleles in gDNA [28]. The averages ± standard deviations of at least two determinations for each point are shown. The dashed lines at 40% and 60% represent the boundaries of what are considered AEI-positive determinations (*i.e.* differences of greater than 20%) using this method of calculation [28]. (A) The percent rs1051730-[C] (%C) allelic expression from *CHRNA3* is shown for all heterozygous individuals tested in the PFC (black symbols; individuals 1 through 6 are represented as a closed square, open square, closed triangle, open triangle, closed circle or open circle, respectively), the Amyg (blue symbols; individuals 7 through 12 are represented as a closed square, open square, closed triangle, open triangle, closed circle or open circle, respectively) or the Nacc (red symbols; individuals 7 through 12 are represented as a closed square, open square, closed triangle, open triangle, closed circle or open circle, respectively). (B) The percent rs16969968-[G] (%G) allelic expression from *CHRNA5* is shown for all heterozygous individuals tested in the PFC (black symbols; individuals 1 through 6 are represented as a closed square, open square, closed triangle, open triangle, closed circle or open circle, respectively), the Amyg (blue symbols; individuals 7, 8, and 10 through 12 are represented as a closed square, open square, and open triangle, closed circle or open circle, respectively) or the Nacc (red symbols; individuals 7 through 12 are represented as a closed square, open square, closed triangle, open triangle, closed circle or open circle, respectively). Note that individual “7” (closed square) remains AEI-positive with strong rs16966968-[G] expression (70–75%) in both the Amyg (blue square) and Nacc (red square).(TIF)

Figure S3 **Concordance between** ***CHRNA5*** **AEI (80:20 %G:%A) and heterozygosity at SNPs rs880395 and rs7164030.** An 852 bp region of the *CHRNA5* distal promoter region (∼13.5 kb upstream of the t.s.s.) containing distal promoter SNPs rs880395and rs7164030 was amplified by PCR and amplicons digested with either *Alu* I (cuts the minor allele at rs880395) or *Dde* I (cuts the minor allele at rs7164030). The *Alu* I and *Dde* I digests are shown. Asterisks (*) indicate the presence of the minor allele product in both digests. Only individual “7” (with arrow) showed the presence of the minor alleles at both of these distal promoter SNPs and also showed *CHRNA5* allelic expression imbalance (see Figure 5B).
(TIF)

Table S1 **PCR and QuikChange**® **Primers and Parameters.**
(XLS)

Table S2 **Lack of correlation between CHRNA3 promoter haplotypes and AEI.**
(XLS)
