# Supplementary figures and images for: In Vitro and Ex Vivo Analysis of CHRNA3 and CHRNA5 Haplotype Expression
Source: PLoS One. 2011 Aug 12;6(8):e23373. doi: 10.1371/journal.pone.0023373 (PMC3155531; doi:10.1371/journal.pone.0023373)

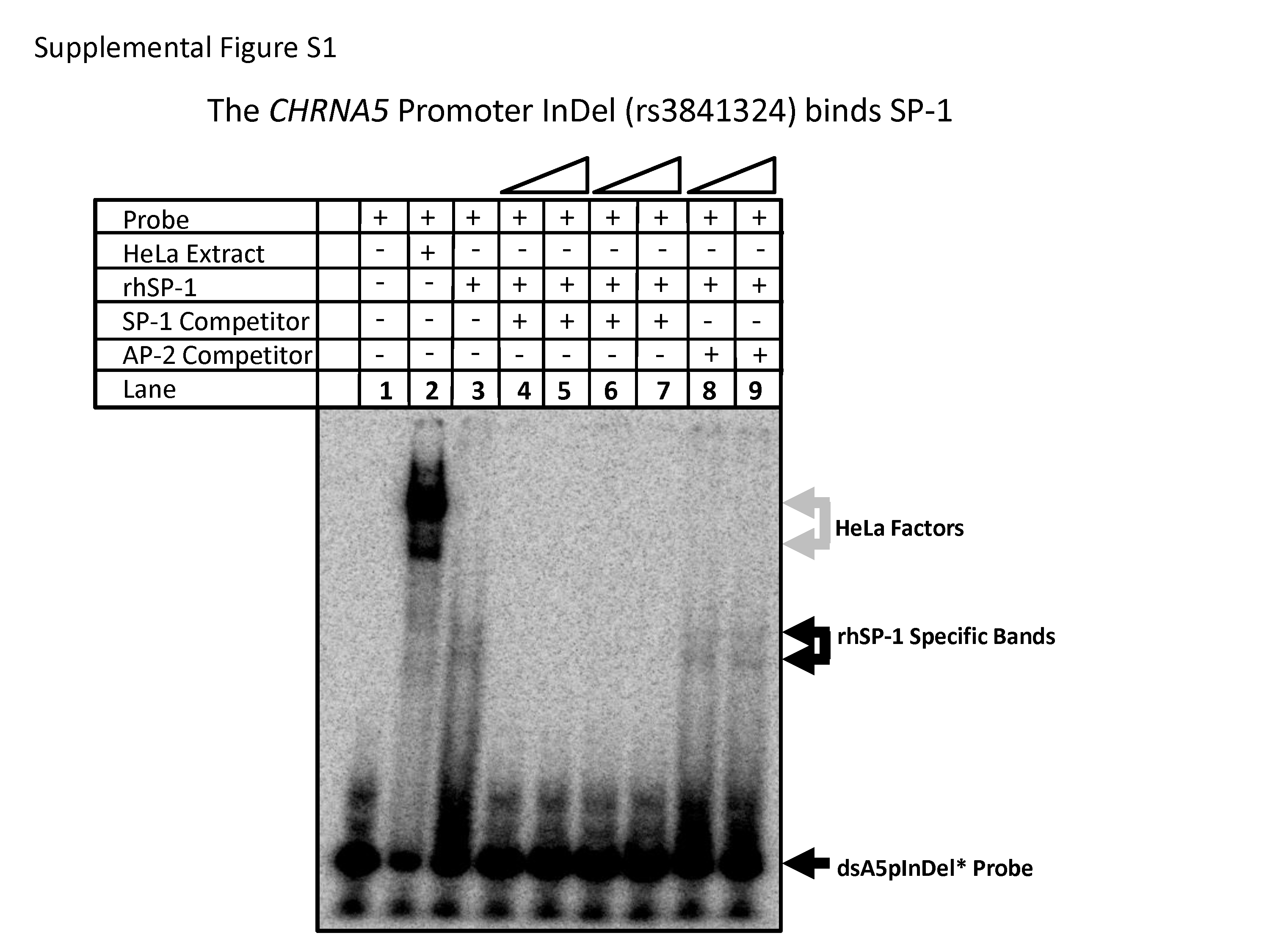

Supplement: Figure S1 — CHRNA5 promoter InDel (rs3841324) sequence binds SP1. Radio-labeled double-stranded oligomers encompassing the CHRNA5 promoter deletion (rs3841324, −227 to −248) were used as probe for binding to 300 ng of pure recombinant human SP-1 (rhSP-1) protein or 10 µg of HeLa nuclear extract. HeLa and rhSP-1 specific bands are indicated by double gray and double black arrows, respectively. The single black arrow indicates the radio-labeled free probe (“dsA5pInDel*”). Right triangles at the top of the schematic indicate lanes with increasing molar excess (50X or 100X) of competitors. Probe alone (lane 1); probe with HeLa nuclear extract only (lane 2); probe with rhSP-1 only (lane 3); probe with rhSP1 and 50 or 100 molar excess of cold probe as competitor (lanes 4 and 5, respectively); probe with rhSP-1 and 50 or 100 molar excess of cold SP-1 consensus oligomer as specific competitor (lanes 6 and 7, respectively); probe with rhSP1 and 50 or 100 molar excess of cold AP-2 consensus oligomer (which, like the SP-1 site, is GC-rich) as non-specific competitor (lanes 8 and 9, respectively). (TIF) [file pone.0023373.s001.tif]

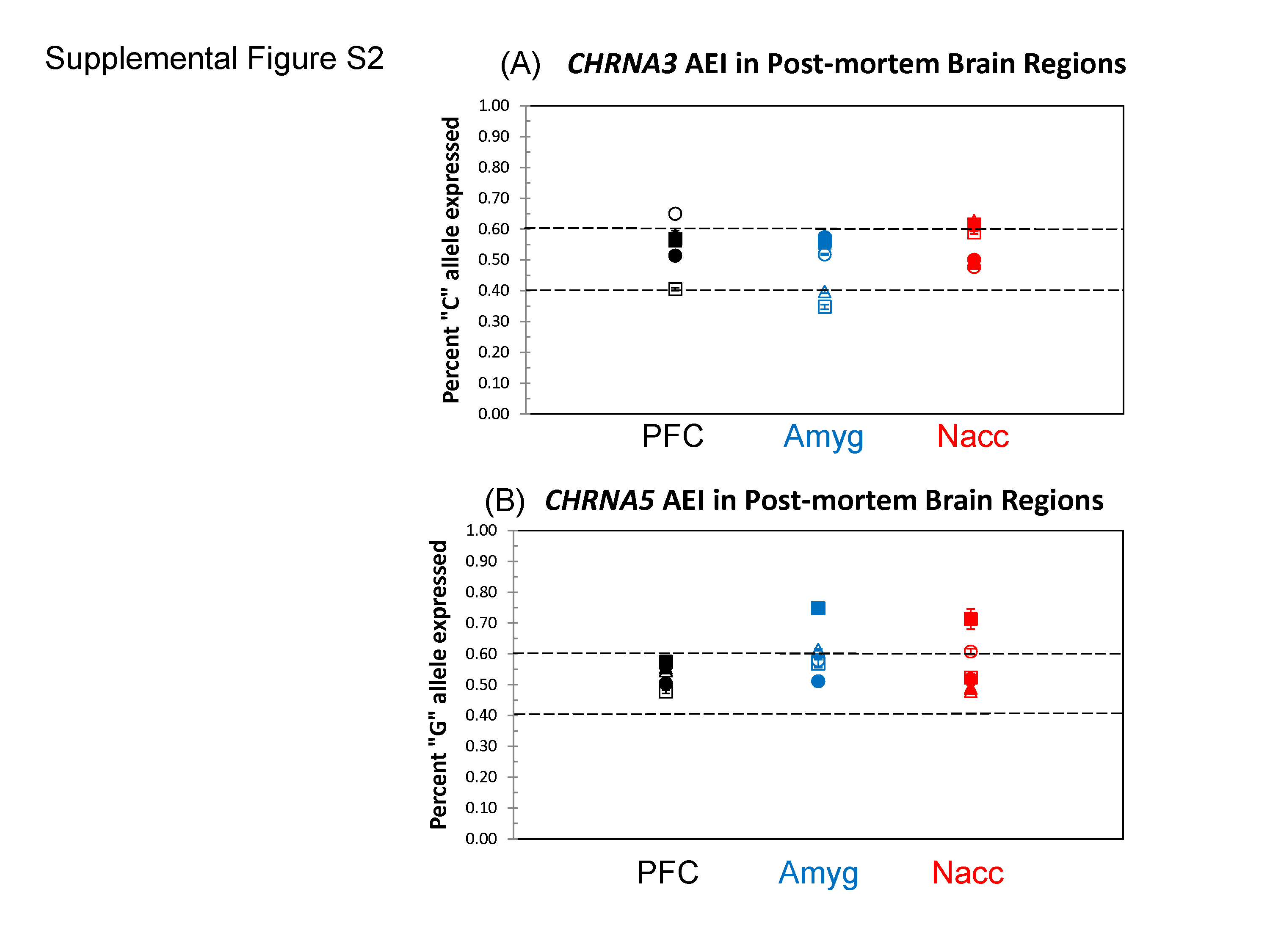

Supplement: Figure S2 — Allelic expression imbalance (AEI) of CHRNA3 and CHRNA5 in post-mortem brain regions. The SNaPshot system was used to assess the relative allelic expression of either CHRNA3 (A) or CHRNA5 (B) in complementary DNA (cDNA) preparations derived from mRNA of post-mortem brain regions, including the prefrontal cortex (PFC, black symbols), amygdala (Amyg, blue symbols) or nucleus accumbens (Nacc, red symbols). Allelic expression was quantified for each sample using the formula: where f(a) is the relative frequency of the ‘a’ allele expression, Ha and Hb are the peak heights of alleles ‘a’ and ‘b’ in cDNA and k corrects for the unequal amplification of SNP alleles in a heterozygous gDNA sample and is equal to ‘a/b’ where ‘a’ and ‘b’ are the peak heights of the two alleles in gDNA [28]. The averages ± standard deviations of at least two determinations for each point are shown. The dashed lines at 40% and 60% represent the boundaries of what are considered AEI-positive determinations (i.e. differences of greater than 20%) using this method of calculation [28]. (A) The percent rs1051730-[C] (%C) allelic expression from CHRNA3 is shown for all heterozygous individuals tested in the PFC (black symbols; individuals 1 through 6 are represented as a closed square, open square, closed triangle, open triangle, closed circle or open circle, respectively), the Amyg (blue symbols; individuals 7 through 12 are represented as a closed square, open square, closed triangle, open triangle, closed circle or open circle, respectively) or the Nacc (red symbols; individuals 7 through 12 are represented as a closed square, open square, closed triangle, open triangle, closed circle or open circle, respectively). (B) The percent rs16969968-[G] (%G) allelic expression from CHRNA5 is shown for all heterozygous individuals tested in the PFC (black symbols; individuals 1 through 6 are represented as a closed square, open square, closed triangle, open triangle, closed circle or open circle, respectively [file pone.0023373.s002.tif]

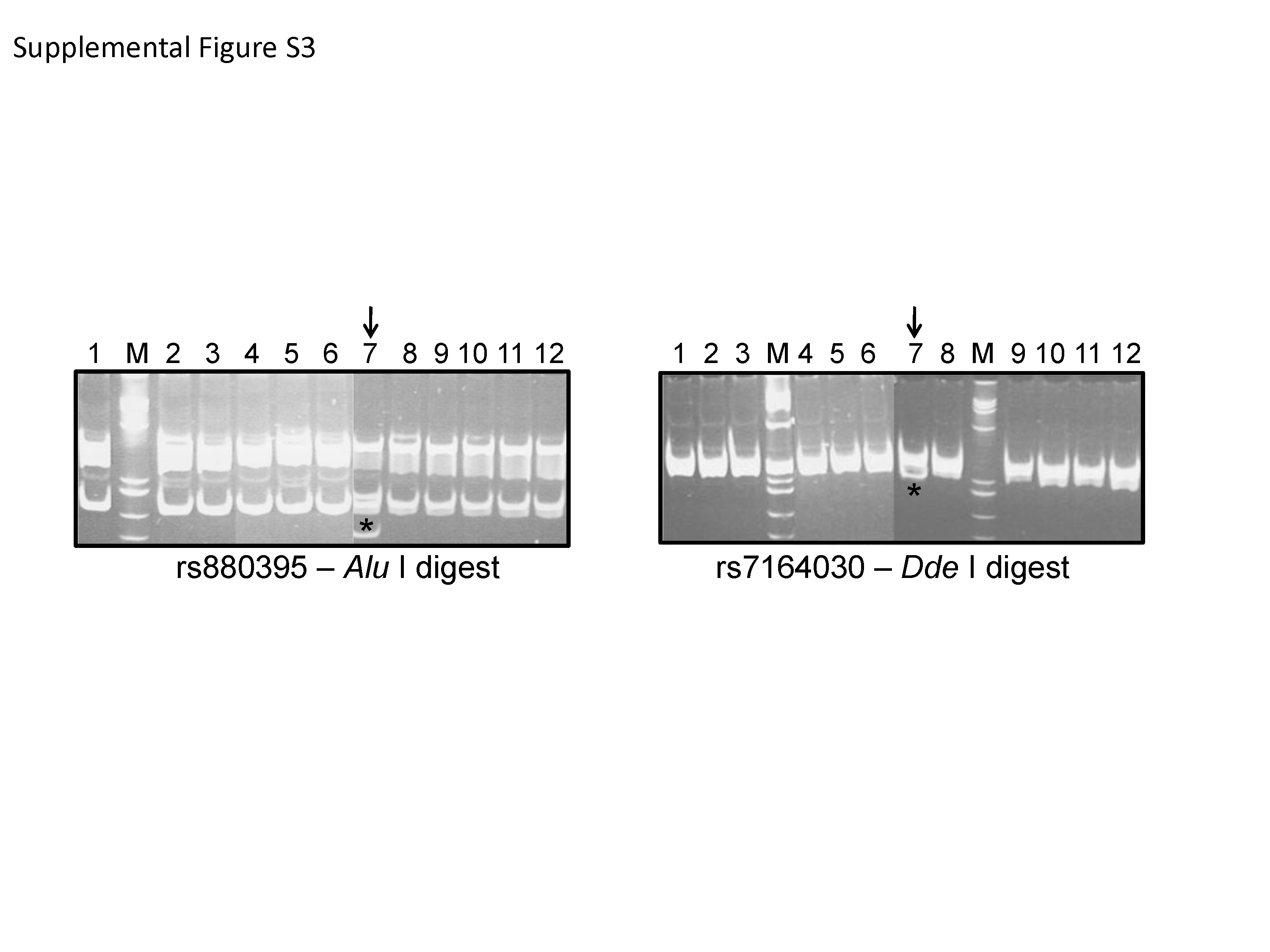

Supplement: Figure S3 — Concordance between CHRNA5 AEI (80:20 %G:%A) and heterozygosity at SNPs rs880395 and rs7164030. An 852 bp region of the CHRNA5 distal promoter region (∼13.5 kb upstream of the t.s.s.) containing distal promoter SNPs rs880395and rs7164030 was amplified by PCR and amplicons digested with either Alu I (cuts the minor allele at rs880395) or Dde I (cuts the minor allele at rs7164030). The Alu I and Dde I digests are shown. Asterisks (*) indicate the presence of the minor allele product in both digests. Only individual “7” (with arrow) showed the presence of the minor alleles at both of these distal promoter SNPs and also showed CHRNA5 allelic expression imbalance (see Figure 5B). (TIF) [file pone.0023373.s003.tif]
